# Forced-choice decision-making in modified trolley dilemma situations: a virtual reality and eye tracking study

**DOI:** 10.3389/fnbeh.2014.00426

**Published:** 2014-12-16

**Authors:** Alexander Skulmowski, Andreas Bunge, Kai Kaspar, Gordon Pipa

**Affiliations:** ^1^E-Learning and New Media, Institute for Media Research, TU ChemnitzChemnitz, Germany; ^2^Department of Philosophy, University of NottinghamNottingham, UK; ^3^Social and Media Psychology, Department of Psychology, University of CologneCologne, Germany; ^4^Neuroinformatics, Institute of Cognitive Science, University of OsnabrückOsnabrück, Germany

**Keywords:** virtual reality, decision-making, trolley dilemma, gender, eye-tracking, emotion induction, arousal, affective responses

## Abstract

Based on the frameworks of dual-process theories, we examined the interplay between intuitive and controlled cognitive processes related to moral and social judgments. In a virtual reality (VR) setting we performed an experiment investigating the progression from fast, automatic decisions towards more controlled decisions over multiple trials in the context of a sacrificing scenario. We repeatedly exposed participants to a modified ten-to-one version and to three one-to-one versions of the trolley dilemma in VR and varied avatar properties, such as their gender and ethnicity, and their orientation in space. We also investigated the influence of arousing music on decisions. Our experiment replicated the behavioral pattern observed in studies using text versions of the trolley dilemma, thereby validating the use of virtual environments in research on moral judgments. Additionally, we found a general tendency towards sacrificing male individuals which correlated with socially desirable responding. As indicated by differences in response times, the ten-to-one version of the trolley dilemma seems to be faster to decide than decisions requiring comparisons based on specific avatar properties as a result of differing moral content. Building upon research on music-based emotion induction, we used music to induce emotional arousal on a physiological level as measured by pupil diameter. We found a specific temporal signature displaying a peak in arousal around the moment of decision. This signature occurs independently of the overall arousal level. Furthermore, we found context-dependent gaze durations during sacrificing decisions, leading participants to look prolonged at their victim if they had to choose between avatars differing in gender. Our study confirmed that moral decisions can be explained within the framework of dual-process theories and shows that pupillometric measurements are a promising tool for investigating affective responses in dilemma situations.

## Introduction

Moral judgments are central to social behavior. We constantly assess our and other’s behavior in moral terms and adjust our actions to what we and our social environment consider to be morally right. In recent years there has been an increasing interest in cognitive and social sciences regarding the cognitive and emotional processes underlying moral judgments. Research in social cognitive psychology provides strong evidence that moral judgments are often driven by quick, automatic, and affectively tinged intuitions (e.g., Fiske et al., 1999; Haidt, 2001), while it is also acknowledged that contextual factors determine whether automatic emotional responses or controlled cognitive responses prevail in a given situation (e.g., Greene et al., 2001). Usually these judgment mechanisms are investigated by letting participants respond to construed moral dilemmas. These dilemma situations can be divided into personal and impersonal dilemmas based on the level of personal involvement (Greene et al., 2001). While personal dilemmas involve the immediate application of force (e.g., pushing an overweight man off a bridge in order to stop a trolley from colliding with five people), impersonal dilemmas do not involve direct physical contact and are therefore rather remote interactions (such as changing the direction of a trolley from the distance by pushing a lever and thereby sacrificing one person in order to save five). The primary objective of the present study is to validate the use of immersive virtual reality (VR) technology in moral dilemma studies by replicating the results obtained in previous studies using textual presentations of the trolley dilemma. In the following paragraphs we will summarize the past research on the trolley dilemma, explain the benefits of employing virtual environments, and elaborate in more detail on the rationale for our study, including our use of additional measurements (eye-tracking and pupillometry) as well as the employment of additional experimental conditions (one-against-one-dilemmas).

The trolley dilemma was originally devised as a philosophical thought experiment (Foot, 1978; Thomson, 1985) and has since been utilized in various psychological studies to shed light on moral judgment and decision-making (e.g., Greene et al., 2001, 2004; Valdesolo and DeSteno, 2006; Hauser et al., 2007). In the original version of this dilemma, one is asked to imagine someone driving a trolley headed for five people standing on tracks who will be killed if the trolley proceeds on its present course. It is not possible to stop the trolley, but it can be turned onto an alternate set of tracks where it will kill one person instead of five (Foot, 1978). Thomson (1985) developed a modified version of the dilemma in which one is not the driver of the trolley but a bystander standing near the tracks who has the opportunity to hit a switch in order to turn the trolley. The question in both versions is whether it is morally right to turn the trolley. Researchers such as Greene (2008) consider the trolley dilemma to be a paradigmatic case in which cognitive responses predominate due to the impersonal nature of the situation. Impersonal dilemmas lead most people to exert a *utilitarian* (or, more broadly, *consequentialist*) judgment: they tend to bring about the best overall consequences at the cost of the well-being of single individuals. Several psychological studies using variants of this dilemma have shown that a vast majority of people tend to endorse the alternative conforming to utilitarianism, i.e., they sacrifice one person to save five (e.g., Valdesolo and DeSteno, 2006; Hauser et al., 2007). However, in comparable personal dilemma situations that require direct physical force to sacrifice the single person, people tend to be more passive and let the five people die. As an explanation, Greene proposes a dual-process theory according to which both automatic emotional responses and more controlled cognitive responses play crucial roles in moral judgments (Greene, 2008; Greene et al., 2008). More specifically, he argues that utilitarian moral judgments are driven by controlled cognitive processes while non-utilitarian judgments are driven by automatic emotional responses. The trolley dilemma predominantly triggers controlled processes, while dilemmas involving the application of direct physical force trigger affective responses in a stronger manner.

Previous research on moral decisions within the trolley paradigm has been primarily carried out using written scenario descriptions (sometimes supplemented by an image depicting the scene). This has led to the negligence of important dynamic situational and emotional aspects of this dilemma. A written and static version of the scenario will most likely not feature any dynamics in visual presentation that elicit emotional arousal. This not only applies for pen-and-paper versions but also for fMRI studies investigating the trolley dilemma (e.g., Greene et al., 2001; Harenski and Hamann, 2006). Accordingly, Patil et al. (2014) emphasized that “the advantage of relying on text- or graphic-based questionnaires is its great experimental controllability, but the downside is that it greatly simplifies the issue at hand by removing all the nonessential contextual features of the dilemmas, raising issue of generalizability of the obtained results” (p. 95). Hence, previous studies could have systematically overestimated cognitive processes compared to emotional ones (cf. Bzdok et al., 2012). Furthermore, most previous studies included only one trial, thus leading to a one-shot study design that fails to measure within-subject changes in moral decisions due to repeated presentations and possible influences of explicit rationalization after having made a decision. A recent study by Friedman et al. (2014) showed that repeated presentations of identical moral scenarios are a suitable approach to investigate potential changes in decision making processes. Despite producing clear results, questionnaire studies only offer a very low degree of immersion. Moreover, current theories of cognition stress the importance of situative and contextual factors (Niedenthal et al., 2005). All of these shortcomings can be remedied by using a VR version of the trolley dilemma.

An emerging trend in social cognition research is to conduct experiments using VR technology in order to elicit more realistic experiences (Bunge and Skulmowski, 2014). The types of thoughts and emotions that would be evoked in real life are likely to be generated during the experiment, proving the ecological validity of this method (Rovira et al., 2009). Virtual reality allows us to conduct experiments that would be ethically unacceptable to execute in non-virtual environments. Several studies have shown that participants’ subjective, behavioral, and physiological responses in VR environments map their behavior and experience in real world settings, such as in the VR replication of Milgram’s (1963) obedience experiment by Slater et al. (2006). When the interaction possibilities mirror real actions, virtual environments provide a feeling of immersion or presence (Carassa et al., 2005). Furthermore, additional equipment can be linked to the VR device in order to record physiological measures of emotional arousal.

Following a pilot study by Pan and Slater (2011), a study by Navarrete et al. (2012) was the first attempt to use the trolley dilemma in a VR setting for moral cognition research. In this study, each participant was standing on a platform overhanging a railway track splitting up into two tracks. Directly in front of the participant there was a rail switch enabling participants to lead a boxcar rushing down the main track onto the side track. Participants had to decide whether they want to change the direction of the boxcar in order to save a group of virtual persons (avatars) on one track by sacrificing a single avatar on another track. In accordance with a previous large-scale pen-and-paper study (Hauser et al., 2007), Navarrete et al. (2012) found that 90% of the participants endorsed the utilitarian outcome. However, this study suffers from various limitations. Most importantly, it only featured a one-shot study design similar to most previous studies, making the investigation of potential changes in moral decisions across repeated scenario presentations impossible. Additionally, we found the course of action in the virtual environment to be unnecessarily confusing since the avatars were not visible at the time that the boxcar arrives. Furthermore, participants were bystanders observing the scene, leading to an impersonal version of the scenario that usually elicits low emotional responses (Greene et al., 2001). However, it has to be acknowledged that using the impersonal version of the trolley dilemma in VR does lead to considerable emotional engagement. Navarrete et al. (2012) and Patil et al. (2014) showed that emotional engagement in a moral dilemma significantly benefits from the increased degree of immersion elicited by VR.

The present study builds on Navarrete et al. (2012) study and implements several improvements. Instead of our participants being passive bystanders watching the scene from a distance, they were the drivers of the train. Because research on user experiences in VR revealed that a first-person view elicit an even higher involvement and a higher sense of spatial presence than a third-person view as realized by Navarrete et al. (cf. Kallinen et al., 2007), we introduced this change of perspective. Hence, although this change of perspective does not involve the immediate application of force, i.e., a haptic component that is characteristic for personal versions of moral dilemmas and leads to strong emotional responses (cf. Greene et al., 2001), being the driver of the trolley instead of merely seeing the scene from a distance was expected to elicit strong emotional responses as well. Moreover, in contrast to previous studies on the trolley dilemma, we wanted to examine the effect of being repeatedly confronted with a dilemma situation to investigate behavioral changes over time. Two recent VR studies have let participants make moral decisions repeatedly (Friedman et al., 2014; Patil et al., 2014), though they varied the context of the decision (e.g., substituting the trolley with a number of other ways to put virtual people in danger). We chose to present our participants repeatedly with instances of the same dilemma in one session. The response patterns elicited through the repeated presentation of a dilemma reveal possible rationalization processes that occur after a decision has been made. Moreover, they can be used to test assumptions of dual-process models of attitude-guided behavior (Olson and Fazio, 2009). While the first few confrontations with a dilemma should lead to rather spontaneous and automatic responses, during the course of the experiment participants have the necessary time to reflect upon their decision tendencies in an elaborated and controlled manner. Unlike in the usual pen-and-paper studies, these patterns can be investigated in the realistic setting that VR provides. Lastly, although we consider the measurement of arousal via electrodermal activity by Navarrete et al. (2012) to be a good first step, we chose to introduce pupillometry to moral cognition research due to its finer temporal resolution. At the same time, the use of eye tracking equipment allows us to analyze participants’ gaze behavior. Virtual reality equipment also enables the exact and constant timing of decision time frames as well as the measurement of response times, unlike pen-and-paper studies.

Despite these differences in the study design, the strong tendency that participants exhibited in previous studies led us to hypothesize that participants will sacrifice the single person instead of the group independently of how often they were already confronted with the dilemma (hypothesis 1). By replicating and extending the finding that participants tend to act utilitarian in trolley dilemma situations, we also aim to validate our paradigm to use repeated dilemma confrontations from the trolley driver’s perspective in a fully dynamic VR environment.

Virtual reality equipment also enables the presentation and synchronized recording of reactions to other stimuli such as music within the dynamic virtual environment. Seidel and Prinz (2013) showed that negative emotions such as anger, annoyance, and irritation can be induced by playing harsh noise and that this manipulation impacts moral judgments. We hypothesized that annoying noise might also influence people’s moral behavior and people’s assessment of their own behavior. A higher arousal induced by a state of anger, we assumed, might lead to a tendency towards killing more avatars as a way of relieving frustration (hypothesis 2), as state hostility has been shown to correlate with frustration in the context of video game aggression (Anderson and Dill, 2000).

For the three other conditions of the experiment, we introduced a forced-choice one-against-one modification of the trolley dilemma. This decision task occurred at rail junctions at which participants could choose whether to direct the trolley onto the left or right rail (one target person per rail). In this sense, utilitarian judgments such as in the classical trolley dilemma are no longer possible. Instead, specific features of the individual target persons become the basis for moral decisions. Building upon research on attitudes towards gender and ethnic outgroups, we varied the gender of the two persons on the rails and, in another condition, the skin color of the targets. On various implicit measures, people usually show more positive attitudes towards females than towards males (Fazio and Olson, 2003). This is clearly the case for women, but even men, who might be expected to show stronger positive attitudes to their own group, show, if at all, only a minor preference for men (e.g., Nosek and Banaji, 2001; Richeson and Ambady, 2001; Rudman and Goodwin, 2004). Accordingly, we expected that at least our female participants would lean towards sacrificing the male avatar in the first meaningful trial (i.e., a trial involving avatars) of the gender condition, when the dilemma has not been encountered before (hypothesis 3). As for ethnicity, whites show generally more positive automatic attitudes towards whites than towards blacks (e.g., Fazio et al., 1995; Dovidio et al., 1997; Greenwald et al., 1998; Nosek and Banaji, 2001). In a VR setting, Dotsch and Wigboldus (2008) found that native Dutch participants on average kept more distance towards avatars with a Moroccan appearance compared to avatars with a white appearance. We therefore expected that our white participants will exhibit a tendency towards sacrificing the dark skinned avatar at least in the first trial, when the dilemma content is still a surprise (hypothesis 4). Various studies also utilized more explicit measures like basic self-reports, semantic differentials, or feeling thermometers (e.g., Eagly and Mladinic, 1989; Greenwald et al., 1998; Rudman and Goodwin, 2004). Here the findings are not univocal, but there is a general trend among explicit measures to express positive attitudes both towards ingroup members and outgroup members. A dissociation between implicit attitudes and self-reported attitudes is most likely to be observed for socially sensitive issues (Dovidio et al., 1997) and is well explained by the MODE model (Fazio, 1990; Fazio and Olson, 2003; Olson and Fazio, 2009). The acronym MODE stands for *mo*tivation and *o*pportunity as *de*terminants for the attitude-behavior relationship. If the opportunity (e.g., sufficient time) and motivation (e.g., concern about evaluation by others) is given, people reflect on their attitudes and control their response. Hence, we should observe a shift from an initial spontaneous categorization towards socially accepted responses over the course of multiple trials. Other psychological models like the motivated tactician metaphor (Schwarz, 1998) and the continuum model (Fiske et al., 1999) would predict a similar outcome. As the gender and ethnicity conditions involve socially sensitive issues, we expected that participants would be motivated not to show socially inacceptable behavior. Accordingly, we hypothesized that our participants would deliberately balance the amount of males and females as well as the amount of blacks and whites that they sacrifice over the course of the trials (hypotheses 5 and 6, respectively).

For exploratory reasons, we also included an experimental condition in which participants had to choose between sacrificing a person facing away from them and a person facing toward them in which the avatar facing away from them was just a double of the avatar gazing at them that had been rotated by 180° along its longitudinal axis. As face recognition and eye contact are hard-wired cognitive building blocks of social interactions (Emery, 2000), we expected that participants would have fewer scruples about sacrificing the avatar facing away from them than the avatar looking at them (hypothesis 7).

The alternatives in the group dilemma are more obvious (a single avatar vs. a group of avatars) than in situations in which the decision is based on avatar features (male vs. female, black vs. white, and facing vs. facing away). Accordingly, we hypothesized that the response times in the group dilemma would be shorter than in all other dilemmas (hypothesis 8).

Moreover, based on research on pupil diameter change during decision-making (Simpson and Hale, 1969), we hypothesized that there will be a specific time signature displaying a peak in arousal around the moment of decision (hypothesis 9).

Furthermore, studies on the influence of emotionally valenced sounds on pupil diameter (Partala and Surakka, 2003) suggest that our music condition will lead to a greater pupil size as a result of a higher emotional arousal (hypothesis 10).

Finally, gaze behavior in relation to sacrificing decisions in the trolley dilemma has already been investigated in pen-and-paper studies (Kastner, 2010). Kastner found that people avoid looking at pictures of the sacrificed individuals. Accordingly, we expected that participants will avert their gaze from the virtual avatars that they chose to sacrifice (hypothesis 11).

## Materials and methods

### Participants

Sixty-six students (36 female) participated for course credits or payment. The study was described as an experiment concerning decisions in dilemma situations. Ages ranged from 18–30 years with a mean of 21.97 (*SD* = 2.31). To check for possible influences of video game consumption, we asked participants how long they play video games in a post-test questionnaire. On average, participants played video games for 1.92 (*SD* = 4.32) hours per week and 1.21 (*SD* = 2.99) of these hours were spent on video games in which they kill virtual persons, showing that our participants are within the range of people without addictive or other problem video game use (Porter et al., 2010). They had normal or corrected-to-normal visual acuity and were naive to the purpose of the study. Due to pupil recognition problems and shifts of the head-mounted display (HMD), we could analyze the eye tracking recordings of only 41 participants. The present study conformed to the Code of Ethics of the American Psychological Association, to the Declaration of Helsinki, and to national guidelines. Written, informed consent was obtained from all participants. The study was approved by the ethics committee of the University of Osnabrück.

### Stimuli and design

During the experiment, participants were seated in the cockpit of a virtual trolley that was controllable at a rail junction using the arrow keys on a keyboard. In the gender dilemma, one track branched off to the left and another track branched off to the right at the junction. On the middle track, right behind the junction, a train was blocking the track. The participants’ task in this experiment was to avoid a collision with the other train by steering the trolley either onto the left or right track at the junction. A female and a male agent were standing on the two branching tracks. The virtual world in this experiment consisted of a tunnel with a rail track and a gravel ground. We used a tunnel to make the two avatars appear on screen at a fixed time.

In condition 1, each participant was presented with 10 fixed pairs of men and women in a randomized order. The number of trials in which the female and the male avatar were standing on a particular side was counterbalanced within participants. In order to prevent habituation to the decision-task, each condition contained 10 filler trials without avatars. We used the filler trials as a means to reduce the predictability of the trials in order to avoid eliciting thoughtless responses from participants. The sequence of meaningful trials and fillers was randomized for each participant. We included additional experimental conditions in which the avatars differed in ethnicity (condition 2) as well as a condition in which participants had to choose between sacrificing a person facing away from them and a person facing toward them (condition 3). These conditions used the same environment as the gender condition. In the group condition (condition 4), participants were presented with a slightly different setup, one directly based on the original trolley dilemma: there was no other train to avoid on the track on which the trolley was driving and only one alternative track to steer the trolley towards. In a counterbalanced manner, this alternative track was either to the right or to the left to the track on which the trolley was driving initially in each trial. On the middle track, a group of 10 avatars was presented at the same time as a single avatar standing on another track (either to the left or to the right). To control the exact time of stimulus onset, distance fog was used to hide the avatars from view and make them appear grayed out for the first second of the presentation in order to prevent judgments based on individual properties of the avatars instead of their number. We chose to use 10 avatars instead of the usually used five people based on pre-tests in order to make sure that participants see at least five avatars even though the distance fog may obscure parts of the avatars.

Based on Seidel and Prinz (2013), the group dilemma was also used to explore the influence of music-induced emotional states on decision making, i.e., an increase in anger and frustration that will lead to a tendency to sacrifice the group. Furthermore, we tested whether the induction of negative emotions leads to a different assessment of participants’ own decisions. We used the harsh and dissonant soundtrack *Inner Mind Mystique I* by Yamazaki “Maso” (Takushi (1996), track 1), which has been shown to elicit negatively valenced emotions. In the 10 meaningful trials of this condition, a fixed group of 10 avatars appearing in the middle were accompanied by 1 of 10 avatars not included in that group appearing alone on another track in a randomized order. As before, 10 filler trials alternated in random order with the meaningful trials.

### Apparatus and setup

Participants were tested individually, and were seated in a darkened room containing two Dell Precision T7500 workstations used for stimulus presentation and eye tracking control. The virtual world was presented on an NVIS nVisor SX60 binocular HMD at a resolution of 1280 × 1024 at 60 Hz. Eye position and pupil dilation of the left eye were measured at a sampling rate of 60 Hz using a monocular ASL EYE-TRAC^®^ 6000 eye tracker with a resolution of 0.25° visual angle. To calibrate, participants fixated on a grid of nine calibration points presented on the HMD. Presentation of the virtual world was performed using the Vizard VR Software Toolkit. The viewpoint had a field of view of 40°. Head-tracking was not used in order to be able to record and analyze eye tracking data.

### Procedure

Upon arrival, participants were first informed about the content and course of the experiment and had to sign an informed consent form acknowledging the right of the participant to end the experiment at any time without any negative consequences. It was pointed out that the experiment involves decisions in social dilemma situations that pertain to the well-being of virtual persons and that music could be played during the experiment. After sitting down, getting the HMD adjusted to their head dimensions and completing the calibration process described above, participants provided demographic and personal information (age, sex, and handedness), were handed a keyboard and were presented with detailed instructions for condition 1 to 3 in German (English translation follows):

“In the following, you will be the operator of a trolley filled with passengers. You are approaching another fully occupied trolley that has come to a halt behind a junction. Your task is to avoid a serious collision.

You cannot stop the trolley, but you can steer it to the left or right at the junction if you react quickly enough. To change the direction of the trolley, push the arrow key of the corresponding direction. A beep sound will indicate when pressing the button is effective. You only have a limited time frame available. Once you have pushed a button, this decision is irreversible and the trolley will change to the corresponding track.

Press the space bar to proceed”.

**Figure 1:**
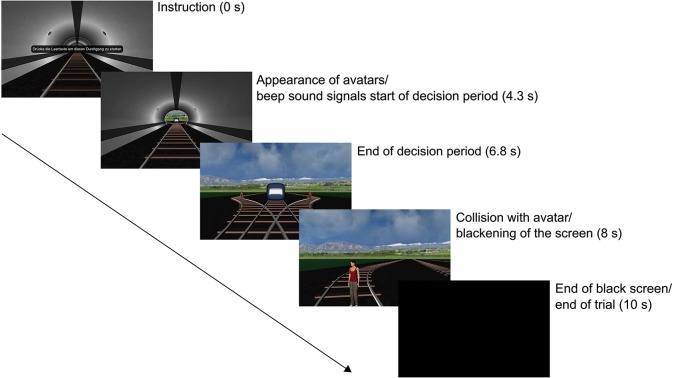
**Overview of the experimental sequence in conditions 1–3**.

The experiment started with six learning trials in which the participants learned the controls and were able to familiarize themselves with the timing. No avatars were present in these trials. Next, the experimental conditions began. The order of conditions 1 to 3 (using avatars of differing gender, ethnicity, and body orientation) was randomized. Each condition consisted of a randomized sequence of 10 meaningful trials that featured avatars and 10 filler trials without avatars. Every trial contained the same sequence of events (see Figure 1): first, participants had to start the trial and accept the continuation of the experiment by pressing the space bar. Then the participant was driving through the tunnel with a constant speed of 72 km/h. The distance from the train’s initial position to the junction’s origin was approximately 135 m. 4.3 s after the beginning of the trial a beep sound occurred, signaling to the participant that key presses would now be effective. In meaningful trials, simultaneous to the beep sound the avatars standing on the left and right branch became visible. Viewed from the origin of the junction, they were placed 7 m to the left and right and 58.5 m ahead. 2.5 s after the beep sound, the train reached the junction, meaning that the participants had to press the left or right arrow key within this period in order to steer the train onto one of the side tracks. The short time interval requires a fast decision without elaborated thinking about the options. The time interval is sufficiently long to allow for responses that are not merely anticipatory, but is too short for an elaborated decision process—at least during the first trial(s). Pre-tests have shown that participants were unable to reliably recognize the avatar properties in a shorter time frame. Right before the collision with the avatars, 8 s after the onset of the trial, the screen was blackened and stayed black for 2 s. In case no response was given, a message stating that they have collided with the other trolley was displayed, signaling an invalid trial. Invalid trials were not repeated in order to keep the time course of the experiment comparable between subjects.

**Figure 2:**
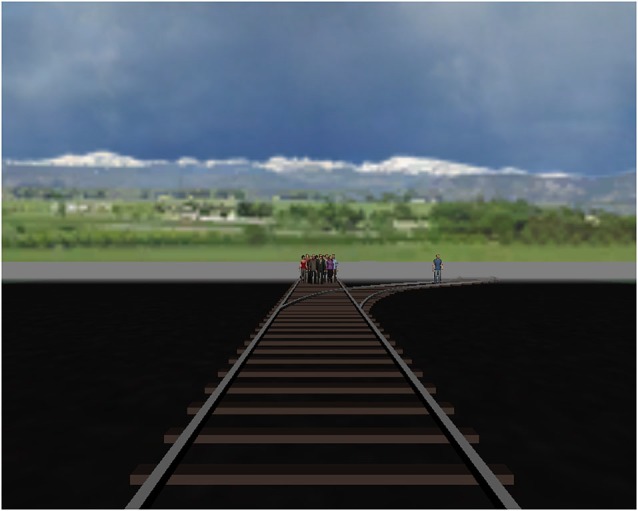
**Screen shortly after the avatars on the rails appeared**.

After finishing the first three conditions, participants took a short break. Since the group dilemma (ten-to-one, condition 4) utilized a slightly different task and environment, all participants were shown new instructions. As this experiment also featured a music-induced emotion manipulation, it was always presented as the last part. Those participants who were assigned to the music condition could hear the irritating music starting from the moment that the new instructions were displayed. Again, six learning trials followed in which no avatars were present. These trials enabled participants to acknowledge the possibility of not steering the trolley by not pushing any button and thereby driving straight ahead. Other than the lack of a tunnel and having only one branching track, the procedure and controls remained the same as for the previous experimental conditions. In the meaningful trials, the group was standing 58.5 m ahead of the junction’s origin. The single person was located either 7 m to the right or to the left of the group (see Figure 2).

Participants in the music condition heard the irritating track for at least 1 min before having to make their first decision involving avatars. After completing the questionnaire described in the following paragraph, participants were debriefed, paid and thanked. The entire session including the calibration of the eye tracker lasted 45 min on average, of which 20 min were used for the VR part.

### Questionnaire

Following the VR part of the experiment, participants had to fill out a questionnaire designed to assess their emotional state, their explicit attitudes towards women, men, blacks and whites, as well as the amount of (violent) video games they play. In addition, participants completed the SES-17 test (Stöber, 1999, 2001) to measure their tendency towards giving socially desirable responses.

In order to investigate whether music can be used to induce emotions within VR experiments, we included the manipulation check introduced in Seidel and Prinz (2013). It consisted of four negatively valenced emotional states (angry, irritated, annoyed, and gross). To further test the effect of music, we also included a positively valenced emotion term (delighted). Following Seidel and Prinz (2013), we asked participants to indicate what kind of emotions they are experiencing “right now.” For each of the five items, they had to respond on a scale ranging from “very little felt” (1) to “very strongly felt” (7). To see whether the music-induced emotional state leads to a stricter judgment of their decisions during the experiment, participants were asked how satisfied they are with the decisions they made in the last condition on a scale ranging from “very satisfied” (1) to “very unsatisfied” (7).

In order to assess participants’ explicit attitudes towards women, men, whites, and blacks we followed Eagly and Mladinic (1989) in using semantic differential scales. Participants were asked to rank each attitude object on five 7-point scales, with the dimensions good-bad, positive-negative, valuable-useless, pleasant-unpleasant, and nice-awful. Each scale was scored from +3 to −3 and attitudes were represented by each participant’s mean response on the five scales. Cronbach’s *α* exceeded 0.80 for each attitude object.

To check for the influence of excessive consumption of violent video games, participants were asked how many hours per week they play video or computer games and how many hours of this time are used to play games in which virtual people are killed. Moreover, in order to measure the tendency of participants towards responding in a socially desirable manner, we included the SES-17 test consisting of 16 self-referred statements (*α* = 0.64) like “In traffic I am always polite and considerate of others” (Stöber, 1999, 2001). Participants had to indicate for each statement whether it is true or false.

### Measurement and preprocessing of pupillary data and gaze duration

Analysis of pupillary data was performed on the time interval between the beginning of the beep sound (=* t1*, coinciding with the appearance of the avatars) and the collision with the avatar (= *t2*). Raw eye tracking data was processed by automatically detecting blinks and removing the resulting artifacts. This was accomplished by discarding the eye tracking data 100 ms before and after a pupil diameter of 0 mm (indicating a blink). Pupil data was interpolated linearly for blinks and tracking losses (after artifact removal). For a similar procedure, see van Rijn et al. (2012). To facilitate the comparison between participants, pupillary data (measured in arbitrary units by the eye tracker and converted to millimeters following the manufacturer’s instructions) was normalized by subtracting the individual participant’s baseline from every recorded diameter of that participant. The baseline was calculated by averaging all pupil diameter measurements during the time from *t1* to *t2* in all 92 trials.

In order to analyze gaze duration towards sacrificed and non-sacrificed avatars, we used the entire left and right half of the screen as areas of interest during the time period between the beep sound and the key press. We chose these areas in order to include any searches for additional clues surrounding the avatars in our analysis and to account for any additional fixations that occurred as a consequence of the fact that the avatars’ on-screen position kept moving during presentation.

## Results

### Behavioral results

During the experimental procedure participants were confronted with four dilemma situations in which avatars differing in either gender, ethnicity, body orientation, or number were present. In conditions 1 to 3, only around 2% of the trials involving avatars were invalid, due to a lack of reaction leading to a collision with the trolley on the middle track. We considered these rare trials as irrelevant and excluded them from further analysis. For each experimental condition we were interested in how many avatars of one group (e.g., males) were sacrificed compared to the number of avatars of the other group (e.g., females). The overall percentages across all 10 meaningful trials are summarized in Table 1.

**Table 1:** **Percentages of sacrificed avatars for each experimental condition**.

Condition	All participants (*n* = 66)		Female participants (*n* = 36)		Male participants (*n* = 30)	
	% (SD)	95% CI	% (SD)	95% CI	% (SD)	95% CI
Gender: sacrificed males	58.28 (18.89)	[53.64, 62.93]	55.16 (17.39)	[49.28, 61.04]	62.03 (20.20)	[54.48, 69.75]
Ethinicity: sacrificed whites	49.04 (15.23)	[45.29, 52.78]	50.79 (17.13)	[45.00, 56.99]	46.93 (12.55)	[42.24, 51.61]
Body direction: sacrificed facing avatars	48.19 (27.99)	[41.30, 55.07]	46.17 (28.36)	[36.58, 55.77]	50.60 (27.84)	[40.21, 61.00]
Group: sacrificed singles	95.67 (14.47)	[92.20, 99.32]	96.11 (10.22)	[92.65, 99.57]	95.33 (18.52)	[88.42, 100.02]

*Note. Percentages indicate how often the type of avatar mentioned in the first column was sacrificed. A value of 50% would reflect that both avatar types in the respective comparison were sacrificed to an equal degree. Trials in which participants did not respond were excluded from analysis. CI = confidence interval*.

#### Group condition (hypotheses 1 and 2)

**Figure 3:**
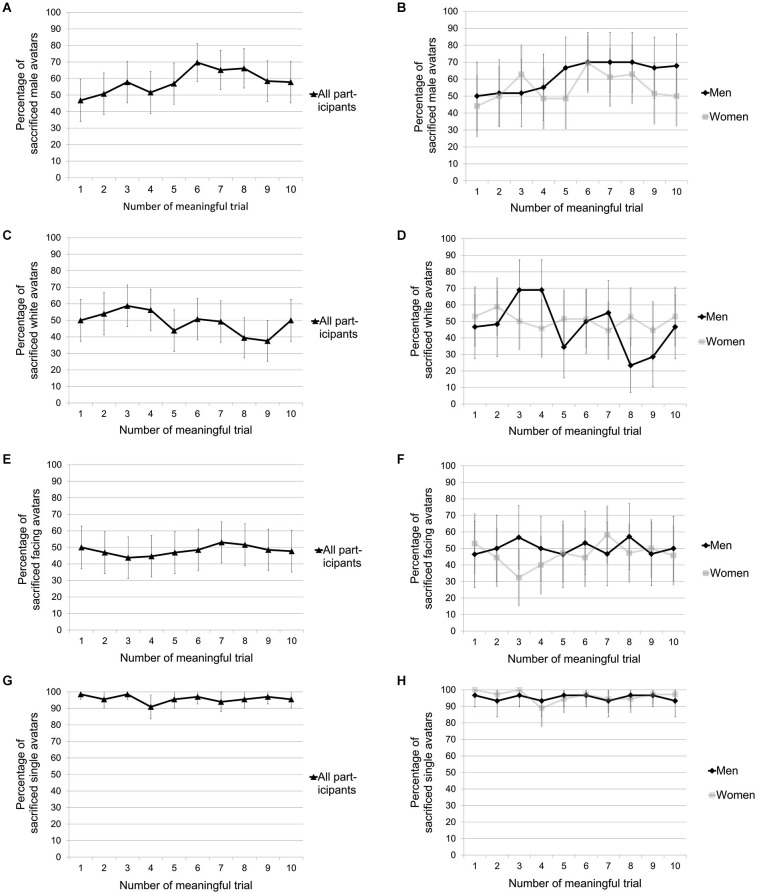
**Percentages of sacrificed avatar types as a function of meaningful trials within condition**. **(A)** Percentage of sacrificed men across 10 meaningful trials in the gender condition. **(B)** Percentage of sacrificed men across 10 meaningful trials in the gender condition separated by participants’ gender. **(C)** Percentage of sacrificed whites across 10 meaningful trials in the ethnicity condition. **(D)** Percentage of sacrificed whites across 10 meaningful trials in the ethnicity condition separated by participants’ gender. **(E)** Percentage of sacrificed avatars facing towards the participant across 10 meaningful trials in the body direction condition. **(F)** Percentage of sacrificed avatars facing towards the participant across 10 meaningful trials in the body direction condition separated by participants’ gender. **(G)** Percentage of sacrificed singles across 10 meaningful trials in the group condition. **(H)** Percentage of sacrificed singles across 10 meaningful trials in the group condition separated by participants’ gender. Error bars represent 95% confidence intervals.

The most substantial effect was detected in the group dilemma (condition 4). Here, participants decided in 96% of the cases to sacrifice the single person in order to save the group, confirming our first hypothesis. Both male and female participants showed this clear pattern (see Figures 3G,H). Fifty-five of the 66 participants sacrificed the single person in all of the 10 trials and there was only one participant who sacrificed the group in all trials. We computed a three-way repeated measures ANOVA (Greenhouse-Geisser applied) with gender (2 levels: male vs. female) and music (2 levels: music on vs. music off) as between-subject factors and trial (10 levels) as within-subject factor. No significant main effects or interactions could be detected, all *p* ≥ 0.169, *η*^2^ ≤ 0.02. The absence of a difference in the proportion of sacrificed single persons between the music condition (95.3%) and the silent condition (96.3%) contradicts hypothesis 2. A difference in the participants’ satisfaction with their decision between the music condition (*M* = 0.38, *SD* = 1.74) and the silent condition (*M* = 0.34, *SD* = 1.88) was absent as well, *t*_(64)_ = 0.09, *p* = 0.931, *d* = 0.02. We created a composite score of negative emotions by computing the mean across “annoyed”, “angry”, “irritated” and “gross”. The difference between this score in the music condition (*M* = 2.30, *SD* = 1.22) and the score in the silent condition (*M* = 1.84, *SD* = 0.80) was significant by trend, indicating a stronger negatively valenced mood in the group that heard music, *t*_(57)_ = 1.82, *p* = 0.074, *d* = 0.48. Also by trend, participants in the music group were more delighted than those participants in the control condition, *t*_(63)_ = 1.97, *p* = 0.054, *d* = 0.50.

#### The effect of gender, ethnicity, and body orientation in one-against-one scenarios (hypotheses 3 to 7)

With respect to the gender condition (condition 1), our third hypothesis regarding the female participants’ tendency towards killing more male avatars at least in the first trial has not been confirmed. Only 44% females sacrificed the male avatar in the first trial, a ratio that is not significantly different from 50%, *t*_(33)_ = 0.68, *p* = 0.501, *d* = 0.12. Both genders sacrificed approximately as many men as women during the first five trials in the gender condition (see Figure 3A). Starting with the sixth trial a tendency for sacrificing more men than women emerged. This difference is most pronounced in trials 6 through 8. As can be seen in Figure 3B, the tendency to sacrifice more men than women in the later trials was especially due to the responses of male participants. From trial 5 on, the percentage of men who killed the male avatar remained constant at around 70%. Female participants did not show such a univocal pattern. To further investigate this pattern, we performed a two-way repeated measures ANOVA with gender of participant (2 levels: male vs. female) as between-subject factor and trial number (10 levels) as within-subject factor. It revealed a marginally significant main effect of gender on the frequency of sacrificed men, *F*_(1,52)_ = 3.07,* p* = 0.086, *η*^2^ = 0.06. However, neither an effect of trial number nor an interaction between the number of trial and participants’ gender was detected, all *F*_(9,468)_ ≤ 0.91, *p* ≥ 0.521, *η*^2^ ≤ 0.02. Subsequently, we decided to conduct comparisons based on the averaged sacrificing tendencies across all trials. No clear difference between male (62%) and female (55%) participant’s tendency to sacrifice male avatars could be detected, *t*_(64)_ = 1.48, *p* = 0.143, *d* = 0.37. However, we were mainly interested in comparing the responses with the assumed chance level of 0.5 derived from the null hypothesis that people do not care which avatar they sacrifice. Contradicting hypothesis 5, a one-sample* t*-test comparing the number of sacrificed male avatars (58%) across all meaningful trials with chance level (50%) revealed that significantly more men were sacrificed than women, *t*_(65)_ = 3.56, *p* = 0.001, *d* = 0.44. The deviation from 50% was particularly strong for male participants, *t*_(29)_ = 3.26, *p* = 0.003, *d* = 0.60, and less pronounced for female participants, *t*_(35)_ = 1.78, *p* = 0.084, *d* = 0.30. We can summarize that there is a clear tendency to sacrifice male individuals, but we yielded ambiguous results with respect to the impact of the participant’s gender on the sacrificing decisions. The repeated measures ANOVA and the comparison of the averaged sacrifice tendencies with chance level hint at a tendency of male participants to sacrifice more men as compared to female participants. However, when we compare the average sacrificing tendencies of male and female participants directly, the difference does not reach significance.

Semantic Differential Scales in the questionnaire were used to measure the attitudes of the participants towards women and men. To compare the favorability of the participants’ attitudes towards women and men, we performed a two-way repeated measures ANOVA with gender of participant (2 levels: male vs. female) as between-subject factor and gender of attitude object (2 levels: male vs. female) as within-subject factor. Women (*M* = 1.51, *SD* = 0.99) were evaluated more favorably than men (*M* = 1.35, *SD* = 0.92), *F*_(1,62)_ = 8.90, *p* = 0.004, *η*^2^ = 0.13. However, no effect of participants’ gender could be detected, *F*_(1,62)_ = 0.13,* p* = 0.719, *η*^2^ < 0.01.

We also compared the participants’ behavioral responses with their tendency to present their behavior in terms of social desirability (as measured by the SES-17). After identifying five outliers (data points that were at least 1.5 interquartile ranges below or above the first or third quartile, respectively, on at least one dimension), we decided to calculate Spearman’s correlation statistic, which is less sensitive to outliers than Pearson’s correlation coefficient. Indeed, this led to a significant correlation, *r*_s_ = 0.25, *p* = 0.041, indicating that those individuals who scored high on the SES tended to sacrifice the male avatars instead of female ones. This is an example of how behavior in virtual worlds is influenced by common social norms, underlining the validity of VR experiments for social cognition research.

For the ethnicity and the body orientation condition (see Figures 3C–F) we ran two separate two-way repeated measures ANOVAs with gender of participant (2 levels: male vs. female) as between-subject factor and meaningful trial number (10 levels) as within-subject factor. All main effects and interactions proved to be insignificant, except for a marginally significant interaction between trial number and gender in the ethnicity condition, *F*_(9,459)_ = 1.75, *p* = 0.075, *η*^2^ < 0.03 (see Figure 3D). The sacrificing tendencies in the ethnicity and body orientation conditions were largely balanced. On average each participant sacrificed the white avatars in 49% of the trials in the ethnicity condition, which is not significantly different from 50%, *t*_(65)_ = 0.51, *p* = 0.609, *d* = 0.06. This confirms our hypothesis 6 according to which participants would sacrifice the white and the dark skinned avatar to an equal degree over the course of the condition. In the first meaningful trial of this condition exactly 50% of the participants who gave a valid response sacrificed the black avatar, which contradicts our hypothesis 4, according to which at least in the first trial participants would exhibit a tendency towards sacrificing the dark skinned avatar. Also, body orientation had no influence on participants’ decisions as indicated by the fact that in 48% of the entire meaningful trials participants sacrificed the avatar facing them, which is not considerably different from 50%, *t*_(65)_ = 0.53, *p* = 0.600, *d* = 0.06. Thus, we did not find support for hypothesis 7, according to which participants would sacrifice more avatars facing away from them than those looking at them.

#### Response time comparison (hypothesis 8)

Our hypothesis 8 stated that the response times in the group dilemma will be fastest. Due to a software problem, the recorded response times of some participants were longer than their actual response times. The measuring error differed between participants. We excluded response times above 2.5 s, which was the maximum possible response time, from analysis. This amounts to 6% of the data. Importantly, all results of the reported statistical tests in this section would remain significant when including this data. As predicted, a two-way repeated measures ANOVA (Greenhouse-Geisser applied) including gender (2 levels: male vs. female) as between-subject factor and experimental condition (4 levels: gender condition, ethnicity condition, body orientation condition, and group condition) as within-subject factor reflected a main effect of experimental condition on response time, *F*_(2.22,135.57)_ = 19.88, *p* < 0.001, *η*^2^ = 0.25. An effect of gender was not present, *F*_(1,61)_ = 0.27, *p* = 0.604, *η*^2^ < 0.01. While we can make no safe statements about absolute response times, we can, however, make comparisons between experimental conditions because this is a within-subject factor. Bonferroni adjusted paired comparisons revealed that the gender condition (*M* = 1.42, *SD* = 0.57), the ethnicity condition (*M* = 1.42, *SD* = 0.59) and the body orientation condition (*M* = 1.35, *SD* = 0.57) differed significantly from the group condition (*M* = 1.12, *SD* = 0.45) in mean response time, all *p*s < 0.001, reflecting the fact that participants responded fastest when confronted with the group dilemma. This result suggests that simple utilitarian decisions can be made faster.

### Eye tracking results

#### Pupillary data (hypotheses 9 and 10)

For conditions 1–3, we were interested in whether there was a significant change in pupil dilation over the course of trials (hypothesis 9) and in whether there was a difference in pupil dilation between and within the experimental conditions. As condition 4 featured a slightly different task, a different environment, different lighting conditions, and was always presented last, this condition was analyzed separately.

**Figure 4:**
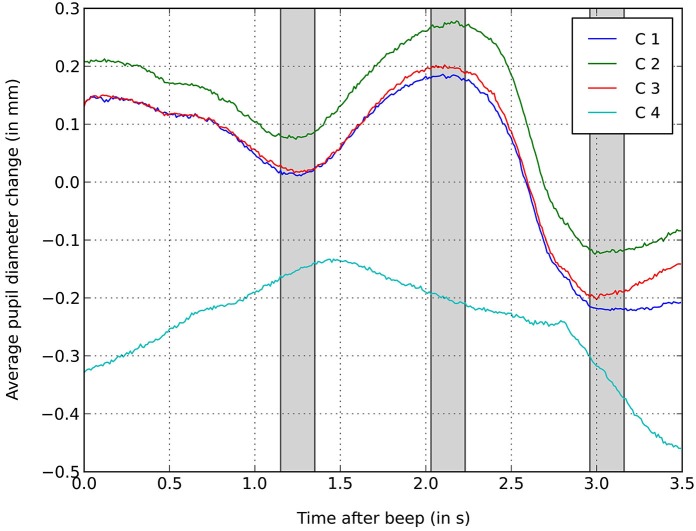
**Averaged relative pupil dilation during the presentation of the avatars**. Plotted separately for each condition (10 trials per participant) and aligned to the onset of the presentation of the avatars at *t* = 0. Pupil dilation is calculated as change relative to the baseline (0).

For the analysis of pupil dilation during the decision process, we considered only those trials that featured avatars. We focused on the time span right after the onset of avatar presentation (coinciding with the beep sound that marked the start of keyboard activation) until the screen was blacked out. The averaged pupil dilations over time (see Figure 4) revealed a characteristic pattern: after a mild decrease in pupil diameter, a global maximum follows which is then succeeded by a high degree of pupil constriction.

For conditions 1 to 3, we selected three bins (as indicated by the gray areas in Figure 4), each with a length of 200 ms, based on the averaged minima and maxima at the described positions. We performed a three-way repeated measures ANOVA with bin (3 levels) as within-subject factor and conditions (3 levels: gender condition, ethnicity condition, body orientation condition) and gender of participant (2 levels: male vs. female) as between-subject factors. We found a significant main effect of bin on pupil diameter, *F*_(2,78)_ = 165.99, *p* < 0.001, *η*^2^ = 0.81. All Bonferroni-adjusted pairwise comparisons between bins were significant, all *p*’s < 0.001. This result confirms our hypothesis 9. Apart from this, no other main effects or interactions were found, all *p* ≥ 0.139, *η*^2^ ≤ 0.05. No effect of gender or condition was found. It is striking that the response times in conditions 1 to 3 coincide fairly well with the beginning increase in pupil diameter at around 1.4 s, indicating a high arousal.

**Figure 5:**
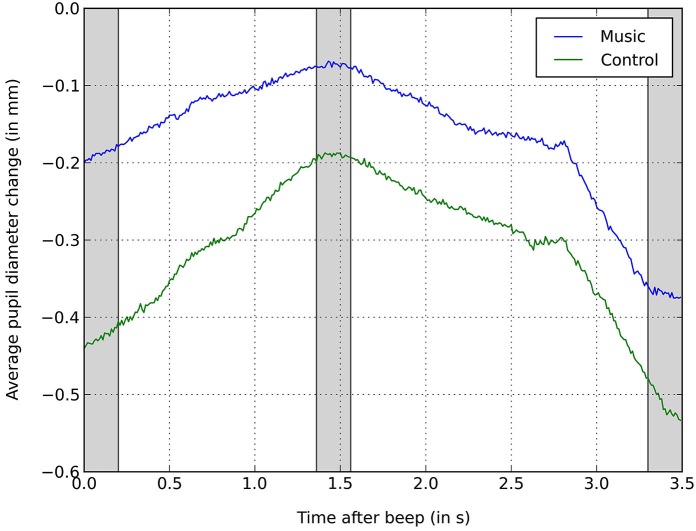
**Averaged relative pupil dilation during the presentation of the avatars during condition 4**. Plotted separately for the music condition and control condition (10 trials per participant each) and aligned to the onset of the presentation of the avatars at *t* = 0. Pupil dilation is calculated as change relative to the baseline (0).

With regards to the pupil dilation during condition 4, we tested whether the music-induced arousal indicated by the increased pupil diameter (see Figure 5) was significant (hypothesis 10). We tested this with another three-way repeated measures ANOVA (Greenhouse-Geisser applied) with bin (3 levels) as within-subject factor and music condition (2 levels: music on vs. music off) and gender (2 levels: male vs. female) as between-subject factors. For this analysis, we chose different bins of the same size matched with the minima and maxima of this condition.

The bins included the 200 ms after the beep, the 200 ms around the global maximum and the 200 ms around the global minimum. Again, there was a main effect of bin, *F*_(1.71,63.26)_ = 60.35, *p* < 0.001, *η*^2^ = 0.62. All Bonferroni-adjusted pairwise comparisons between bins were significant, all *p* ≤ 0.001. Also, we found a main effect of music, *F*_(1,37)_ = 6.67, *p* = 0.014, *η*^2^ = 0.15, confirming hypothesis 10. There was no interaction between bin and music, *F*_(1.71,63.26)_ = 2.453, *p* = 0.102, *η*^2^ = 0.06, indicating that the shape of the pupil dilation curve was similar for the music and control conditions. The higher average pupil diameter in the music condition shows that the irritating music did have an effect on the participants’ arousal, while mixed self-reports (see above) indicated no specific valence. No other significant main effects or interactions were detected, all *p* ≥ 0.102, *η*^2^ ≤ 0.06.

#### Gaze duration for victims and non-victims (hypothesis 11)

We used the eye tracking data to measure the relative time participants spent on looking at the side where the victim was located compared to the time looking at the other side during the decision process (the time period between the beep sound and the key press). We calculated a score for conditions 1 to 3 by dividing the mean time looking at the side where the victim was by the mean time looking at the other side. For this score, a value of 1 reflects a balanced ratio, while a value above 1 indicates that the participant spent more time looking at the side where the victim was located. We found a general tendency across all conditions to spend more time looking at the avatar that is to be sacrificed (*M* = 1.07, *SD* = 0.26), *t*_(40)_ = 1.74, *p* = 0.089, *d* = 0.27. Of all conditions, this effect was strongest for the gender condition (*M =* 1.21, *SD* = 0.65), *t*_(40)_ = 2.08, *p* = 0.044, *d* = 0.32. However, for the ethnicity (*M* = 1.03, *SD* = 0.31) and the body orientation (*M* = 1.07, *SD* = 0.34) conditions this effect was not significant, *p* = 0.558 and *p* = 0.188, respectively. This result implies that the gender dilemma, which featured a particularly sensitive issue, led participants to focus their attention on their victim. While existing research suggests that people will avoid looking at their victims (hypothesis 11), our results show that this gaze tendency is context-dependent. For the group condition the conducted analysis was not feasible, because the group of avatars was, viewed from the participant’s perspective, always straight ahead, rather than on one side or the other.

## Discussion

### Replication of previous results in the group condition

Our 96% rate of utilitarian decisions across the 10 trials in the group dilemma confirms our hypothesis 1. As only one participant decided to sacrifice the group instead of the single avatar in all trials (all other participants stated that they only sacrificed the group accidentally by reacting too late), it is apparent that people stick with their decision and do not change their minds when being confronted with this situation repeatedly. The replication of the result that people tend to make utilitarian choices in trolley dilemma situations and the extension of this result to repeated confrontations with the dilemma validates our paradigm to present participants with moral dilemmas in fully dynamic virtual environments.

### Response times and emotional arousal

We found that the classical one-against-many scenario led to significantly faster response times than the one-against-one dilemmas. In these dilemmas no utilitarian judgments were possible, i.e., subjects had to decide for one of two equally harmful options. Apparently, judgment formation in the group dilemma requires a stronger cognitive elaboration. Moreover, we found evidence that the harsh music increased emotional arousal in the context of the group dilemma. However, we did not find clear evidence for a specific valence. Hence contradicting hypothesis 2, the music did not annoy the participants and did not alter moral decisions. The latter is not surprising, considering that there was little overall variance in participants’ behavior in the group condition. Interestingly, the physiological measure of pupil diameter corresponds to participants’ subjective arousal, while no behavioral change could be observed as a consequence of this affective state. Apparently, the utilitarian action tendency is too strong to be interfered by affective responses. Surprisingly, we found an indication that participants in the music condition might have felt also more delighted than those in the silent condition. This can only be explained by the fact that participants completed the manipulation check after the music had stopped and were asked specifically which emotion they are experiencing right now. Apparently, participants were relieved after the music had terminated. We think it is quite plausible to assume that a feeling of relief might accompany the negative emotional effects of the music that are carried over from the actual experiment.

### The MODE model and its role in gender and ethnicity contexts

Our expectations regarding the gender and ethnicity condition were based on the MODE model by Fazio (1990). One of these expectations was that the first trial of these two conditions would reveal automatic attitude-to-behavior processes. However, there was no such clear tendency in the initial trials. A possible explanation for this is that participants actually have equally positive attitudes towards both genders or both ethnicities and consequently sacrifice these groups in equal amounts. Another plausible explanation would be that the decision time frame was too long to measure implicit attitudes and too short to measure explicit attitudes in the first trial. In order to be able to make clearer distinctions between implicit and explicit processes, future experiments should investigate the effect of varying the decision time frame. For our study, introducing the one-to-one forced choice setting, we opted for a time frame that is long enough for people to recognize the relevant properties, but short enough to prevent controlled cognitive processing.

The MODE model proposes two factors, opportunity and motivation, as determinants of whether a decision is formed in a deliberate or an automatic fashion. Since our participants were confronted with the same dilemma situations numerous times and people generally tend to express balanced views towards outgroups on explicit measures in the case of gender and ethnicity, we expected them to sacrifice these groups in a balanced manner over the 10 trials (hypotheses 5 and 6). In the case of the ethnicity condition this hypothesis was confirmed as the participants sacrificed the avatars of the two kinds to an equal amount over the 10 meaningful trials. However, in the gender condition we observed a clear tendency towards sacrificing male rather than female avatars which contradicts our hypothesis 5 but might still be in line with the MODE model. We assume that participants deliberately sacrificed the male avatars in favor of the female avatars in the later trials because they were motivated to behave in line with a societal norm according to which women have a prior right to preserve physical integrity as compared to men. There is some evidence supporting this interpretation: women were rated more favorably than men in the test for explicit attitudes and, most interestingly, those participants who had a higher tendency to answer in terms of social desirability sacrificed more male avatars. Although not as clearly, we found some indication that the tendency to sacrifice male individuals was strongest for our male participants. Such an effect might be due to a higher societal pressure on men not to privilege men than on women not to privilege their own group. These results are also in line with the motivated tactician metaphor (Schwarz, 1998) that would predict that participants change their behavior into a publicly acceptable manner after becoming aware of the social expectation. Similarly, the continuum model (Fiske et al., 1999) would assume that participants only have the necessary cognitive resources and time to perform re-categorizations and adjust their behavior to social norms in the later trials of each condition. Interestingly, no significant differences could be found in the ethnicity condition. This may be due to the fact that social norm in this context is to treat people of all ethnicities equally. Possibly, our sample may have been more sensitized to social aspects of gender inequality. We therefore suggest to carry out follow-up studies using more diverse samples as well as to conduct this study in different cultural settings.

### Body orientation

Regarding the body orientation condition, our hypothesis (H7) has not been confirmed. Apparently, participants did not have more scruples about sacrificing the avatar facing toward them, possibly because they recognized that there was the same avatar on both tracks. Some participants told us that they sacrificed the avatar facing them since this avatar could see the train approaching and leave the track, while the avatar facing away from them did not have this chance. On the one hand, participants did not want to drive towards the avatar looking right at them, while on the other hand it is considered to be unfair to hurt an avatar that is not aware of any danger. We must however also acknowledge the possibility that these reports merely reflect *post hoc* justifications.

### Pupillary data

From pupillary data we can draw conclusions regarding the cognitive load and emotional involvement in the dilemma situations. In all four conditions, a significant increase in pupil diameter occurred after the moment of decision, indicating participants’ increased arousal or cognitive load at that time. This result confirmed our hypothesis 9. Einhäuser et al. (2010) have shown that pupil dilation is time-locked to moments of decision and that pupil dilation is influenced by post-decisional consolidation of the selected outcome rather than the cognitive appraisal component before the decision.

When examining the pupil diameter elicited in the first three conditions, an increase right after the moment of choice (average response time around 1.4 s in conditions 1–3) stands out followed by a high degree of constriction approximately 1 s after the participants made their decisions. This decrease in pupil diameter is more pronounced in conditions 1 to 3 compared to condition 4, however, since these conditions featured a slight change of luminance (leaving a dark tunnel) starting at the time period we analyzed, this extreme change in dilation needs to be interpreted with caution. However, as a similar pattern featuring a distinct decrease of pupil diameter after the decision was also present in the steadily illuminated environment of condition 4, we can assume that the constriction in condition 1 to 3 was not just an artifact of the change of luminance.

When comparing the conditions, condition 2 (ethnicity) elicited the highest arousal on average, though the difference is not significant (presumably due to the high variance between participants). Nevertheless, we are convinced that emotional reactions to more extreme social decision situations (particularly regarding controversial topics) will prove to be fruitful psychological research venues utilizing immersive VR technology. The increased average pupil diameter in the music condition is in line with our expectation (H10) according to which the noise would lead to higher arousal. It is also in congruence with the higher self-reported emotional arousal (mixed in valence) that we found in the noise condition.

### Gaze duration for victims and non-victims

We found a general tendency across all conditions to spend more time looking at the avatar that will be killed. This tendency was strongest in the gender condition. An eye tracking study that utilized written vignettes and pictures of the scenario found that people generally avoid looking at those individuals that they had sacrificed in a moral dilemma (Kastner, 2010). In contrast to Kastner’s study, which examined viewing behavior after a decision, we focused on participants’ gaze during the decision process. Since our participants did not avert their gaze from their victims and even looked at their victims significantly longer in the gender condition, hypothesis 11 was not confirmed. Our participants may have directed their attention longer at the victim in order to reassure themselves of making a “right” decision. This search for reassurance or clues might be the reason why participants looked longest at their victim in the socially sensitive gender condition.

### Validity of the present study and outlook

In this study we have validated a new research method for studies on moral cognition. Using a fully dynamic VR setup in a repeated confrontation paradigm, we have replicated the findings of previous experiments employing the trolley dilemma in one-shot textual presentation designs. While we replicated the results of non-VR studies, the use of VR offers several important advantages: VR offers a high degree of immersion as a result of the dynamic visual display that cannot be attained with written scenarios. Decision time frames can be held constant in VR studies while participants in pen-and-paper studies read scenario descriptions at varying speed, making comparisons between their response times impossible. Furthermore, we have shown that physiological measurements such as pupillometry can be integrated into VR study designs to measure arousal in addition to self-reports. Additional measures such as heart rate or skin conductance are possible. Our use of eye tracking as a measure for attention could be expanded upon by using more fine-grained regions of interest and by analyzing the temporal dynamics of gaze behavior as well as the formation of established scan-paths over time (cf. Kaspar et al., 2011). In future studies haptic input devices could be used to include sensorimotor data in the VR interaction and to analyze aspects of embodiment.

Since previous studies have found that immersion during VR experiments is high and that responses elicited in these experiments are comparable to those responses found in real-world versions of the experiments (Slater et al., 2006; Rovira et al., 2009), we can assume that our results mirror the behavior that our participants would exhibit in a similar real-world dilemma. In fact, also Patil et al. (2014) emphasized that textual scenario presentation does not include the manifold visual information (but also temporal dynamics) which are accessible to people in real world and hence they cannot access the context-dependent knowledge that is crucial for decision making processes. By applying VR the sensory engagement becomes significantly stronger and the informational scaffold for decision making processes more realistic. Therefore, our study is presumably more ecologically valid than written or static pictorial versions of the corresponding moral dilemmas. Although the situation of steering a trolley and having to decide which person to sacrifice is uncommon, participants immediately understood the dilemma and put effort into their decisions. As already mentioned, they did not perceive it to be an irrelevant video game which makes it likely that their decisions in the virtual environment reflect tendencies that would also be present in the non-virtual world.

Nevertheless, there are also limitations present in our study. First of all, the sensory engagement could be further increased by including a visual representation of the train’s control unit and the participant’s hand, to name just one example. Further limitations concern the comparability to other studies. For instance, the design of the modified trolley scenarios in conditions 1 to 3 does not allow a utilitarian judgment as the classical scenario realized in condition 4, i.e., bringing about the best overall consequences at the cost of the well-being of single individuals. Instead, the moral aspect of these dilemmas is given by the contrasted value assigned to man vs. woman (condition 1), black vs. white person (condition 2), person facing you vs. not facing you (condition 3). Therefore, all choices are equally harmful and unfair on the face of it, considering that in each one person is killed. This makes the study unusual in the field of moral psychology and makes it hard to directly compare this study with the original trolley dilemma. Furthermore, unlike previous VR studies in which participants could refrain from acting (Navarrete et al., 2012; Patil et al., 2014), our participants were forced to make a choice in the one-to-one dilemmas. Forcing participants to kill repeatedly might lead to reduced feelings of responsibility, which in turn might lead to random choices resulting in non-significant results. We have tried to prevent this by introducing filler trials and by varying the dilemma contents throughout the experiment, but cannot completely rule out this possibility.

However, we are convinced that the one-against-one dilemma provides novel important insights into processes of moral judgments as the decision cannot be made simply on the basis of the number of people that would die or can be rescued. Rather this forced-choice situation brings a social comparison into play that refers to different key features of the target person (i.e., gender, ethnicity, and bodily orientation). As a utilitarian judgment is impossible, the dilemmas provoke a decision on the basis of only one salient feature in which the target persons differ. The results show that in general significantly more men were sacrificed than women but that ethnicity and bodily orientation did not affect the decisions. Apparently, some person characteristics elicit a judgment bias while others are not considered (or are consciously counteracted). These findings provide interesting starting points for future research that is necessary to scrutinize the generalizability of the results to other person characteristics.

A further limitation consists, however, in the use of a sample composed of exclusively German university students of white ethnic origin. People from other cultural, ethnic, or educational backgrounds as well as from other age groups may show different reactions. Regarding the external validity, we can therefore only assume that a similar sample will show comparable responses, underlining the importance of cross-cultural follow-up studies using forced-choice dilemmas. As has already been shown, moral judgments can vary greatly between people of different cultural or societal background (Haidt et al., 1993). Another important issue is the chosen length of the decision time frame. Previous studies used very short presentation times of ~200 ms of large static visual stimuli to study implicit attitudes (Payne, 2001; Amodio et al., 2008), which allows for response intervals of less than 500 ms. The decision time we employed might be too long in order to find implicit biases, explaining the lack of significant results in the racism condition and the first few trials in the gender condition. Paradigms like the IAT are superior to detect racial biases because they require a much faster response. In contrast, in this study response times could range up to 2500 ms. The crucial question is whether subjects had enough time to control their initial response biases in favor of a more deliberate decision formation when presented with a particular dilemma for the first time. We argue that this is likely not the case since the time needed to perceive the scene correctly in the here presented dilemmas is much longer. The reason for this is the nature and complexity of the dynamic stimulus used. First, the size of the visual targets was small, i.e., mainly the faces of the persons, which requires saccadic eye movements to each of the targets to perceive the relevant information. Intersaccadic intervals (including the fixation period) are ~350 on average with a very large fraction even longer than 500 ms (Otero-Millan et al., 2008; Dorr et al., 2010). Therefore, a required active exploration of the VR, with one saccade on each target, is expected to last at least 700 ms to more than a second. Hence, the time needed for perception *per se* is much longer than the reported response times of 500 ms that were used in previous studies using static images to investigate fast implicit attitudes. Second, the relevant information in the visual stimuli used there had been rather global, i.e., a white vs. a black face can be decoded purely on the average luminance. This in contrast to the rather small informative patches, i.e., essentially the face of the persons, in this paradigm makes visual processing faster. Lastly, we used fog to allow for a smooth appearance of the visual targets, which led to an ambiguity that slowly faded while the fog lifted, allowing for increased visibility of the relevant targets’ features. This process was taken place on the order of seconds as well. Taken all three effects together, a time needed for pure perception of the scene on the order of 1–2 s is plausible and is experimentally supported by our pre-tests which have shown that people were unable to reliably detect the relevant avatar features when applying shorter decision windows. In conclusion, it appears unlikely that subjects had enough time to control for implicit biases since most of the 2500 ms decision windows were needed for perceptional processes identifying relevant features. Nevertheless, future research using this paradigm should be done varying the decision time frame to investigate whether this has an effect on behavior. It is to expect that the influence of explicit attitudes increases when participants have more time to deliberate. Patil et al. (2014) have chosen a longer decision time frame (10 s), while we were interested in quick, automatic judgments. Especially for our gender condition, a variation of decision time frames could lead to interesting results regarding the interplay between implicit and explicit attitudes.

Finally, a similar VR setting could be used for research on physiological effects of dilemma situations and uncertainty of decisions, e.g., by combining this setup with EEG recordings. Future studies on the interplay between cognitive and affective factors in moral decisions could also focus on more personally involving moral dilemmas that involve physical contact with avatars, possibly implemented by using motion-tracked dummies. Pupillometric studies could focus on differences in the experience of agency on pupil dilation, e.g., by comparing the pupil dilation in our study design with a less immediate mode of interaction, such as only being a bystander at a switch.

## Conclusion

Our VR study confirmed that decisions in dilemma situations can be explained within the framework of dual-process theories such as Greene’s or the MODE model. We were able to replicate the results of previous research on the trolley problem, thereby validating our research method. Additionally, our forced-choice dilemma revealed a general tendency towards sacrificing male individuals, which was associated with socially desirable responding. As indicated by differences in response times, decisions regarding the group dilemma seem to be faster to decide than those based on comparisons of avatar properties such as gender, ethnic origin, or body orientation, since they are based on different moral contents. Furthermore, we were able to manipulate emotion on a subjective and physiological level during decision tasks using music. The specific signature of pupil dilation featuring a peak around the moment of decision is independent of the baseline arousal level.

## Conflict of interest statement

The authors declare that the research was conducted in the absence of any commercial or financial relationships that could be construed as a potential conflict of interest.

## References

[B1] Amodio D. M. Devine P. G. Harmon-Jones E. (2008). Individual differences in the regulation of intergroup bias: the role of conflict monitoring and neural signals for control. J. Pers. Soc. Psychol. 94, 60–74. 10.1037/0022-3514.94.1.60 18179318

[B2] Anderson C. A. Dill K. E. (2000). Video games and aggressive thoughts, feelings and behavior in the laboratory and in life. J. Pers. Soc. Psychol. 78, 772–790. 10.1037/0022-3514.78.4.772 10794380

[B54] Bunge A. Skulmowski A. (2014). “Descriptive & pragmatic levels of empirical ethics: utilizing the situated character of moral concepts, judgment, and decision-making,” in Experimental Ethics, eds Lütge C. Rusch H. Uhl M. (Basingstoke: Palgrave Macmillan). 10.1057/9781137409805.0019

[B3] Bzdok D. Schilbach L. Vogeley K. Schneider K. Laird A. R. Langner R.. (2012). Parsing the neural correlates of moral cognition: ALE meta-analysis on morality, theory of mind and empathy. Brain Struct. Funct. 217, 783–796. 10.1007/s00429-012-0380-y 22270812 PMC3445793

[B4] Carassa A. Morganti F. Tirassa M. (2005). “A situated cognition perspective on presence,” in 27th Annual Conference of the Cognitive Science Society, eds Bara B. G. Barsalou L. Bucciarelli M. (Mahwah, NJ: Erlbaum), 384–389.

[B5] Dorr M. Martinetz T. Gegenfurtner K. R. Barth E. (2010). Variability of eye movements when viewing dynamic natural scenes. J. Vis. 10:28. 10.1167/10.10.28 20884493

[B6] Dotsch R. Wigboldus D. H. J. (2008). Virtual prejudice. J. Exp. Soc. Psychol. 44, 1194–1198 10.1016/j.jesp.2008.03.003

[B7] Dovidio J. F. Kawakami K. Johnson C. Johnson B. Howard A. (1997). On the nature of prejudice: automatic and controlled processes. J. Exp. Soc. Psychol. 33, 510–540 10.1006/jesp.1997.1331

[B8] Eagly A. H. Mladinic A. (1989). Gender stereotypes and attitudes toward women and men. Pers. Soc. Psychol. Bull. 15, 543–558 10.1177/0146167289154008

[B9] Einhäuser W. Koch C. Carter O. L. (2010). Pupil dilation betrays the timing of decisions. Front. Hum. Neurosci. 4:18. 10.3389/fnhum.2010.00018 20204145 PMC2831633

[B10] Emery N. J. (2000). The eyes have it: the neuroethology, function and evolution of social gaze. Neurosci. Biobehav. Rev. 24, 581–604. 10.1016/s0149-7634(00)00025-7 10940436

[B11] Fazio R. H. (1990). “Multiple processes by which attitudes guide behavior: the MODE model as an integrative framework,” in Advances in Experimental Social Psychology (Vol. 23), ed Zanna M. P. (New York: Academic Press), 75–109.

[B12] Fazio R. H. Jackson J. R. Dunton B. C. Williams C. J. (1995). Variability in automatic activation as an unobtrusive measure of racial attitudes: a bona fide pipeline? J. Pers. Soc. Psychol. 69, 1013–1027. 10.1037/0022-3514.69.6.1013 8531054

[B13] Fazio R. H. Olson M. A. (2003). Implicit measures in social cognition research: their meaning and use. Annu. Rev. Psychol. 54, 297–327. 10.1146/annurev.psych.54.101601.145225 12172003

[B14] Fiske S. T. Lin M. H. Neuberg S. L. (1999). “The continuum model: ten years later,” in Dual Process Theories in Social Psychology, eds Chaiken S. Trope Y. (New York: Guilford), 231–254.

[B15] Foot P. (1978). “The problem of abortion and the doctrine of the double effect,” in Virtues and Vices and Other Essays in Moral Philosophy, ed Foot P. (Berkeley: University of California Press), 19–32.

[B16] Friedman D. Pizarro R. Or-Berkers K. Neyret S. Pan X. Slater M. (2014). A method for generating an illusion of backwards time travel using immersive virtual reality—an exploratory study. Front. Psychol. 5:943. 10.3389/fpsyg.2014.00943 25228889 PMC4151165

[B17] Greene J. D. (2008). “The secret joke of kant’s soul,” in Moral Psychology: The Neuroscience of Morality—Emotion, Brain Disorders and Development (Vol. 3), ed Sinnot-Armstrong W. (Cambridge: MIT Press), 35–80.

[B18] Greene J. D. Morelli S. A. Lowenberg K. Nystrom L. E. Cohen J. D. (2008). Cognitive load selectively interferes with utilitarian moral judgment. Cognition 107, 1144–1154. 10.1016/j.cognition.2007.11.004 18158145 PMC2429958

[B19] Greene J. D. Nystrom L. E. Engell A. D. Darley J. M. Cohen J. D. (2004). The neural bases of cognitive conflict and control in moral judgment. Neuron 44, 389–400. 10.1016/j.neuron.2004.09.027 15473975

[B20] Greene J. D. Sommerville R. B. Nystrom L. E. Darley J. M. Cohen J. D. (2001). An fMRI investigation of emotional engagement in moral judgment. Science 293, 2105–2108. 10.1126/science.1062872 11557895

[B21] Greenwald A. G. McGhee D. E. Schwartz J. L. K. (1998). Measuring individual differences in implicit cognition: the implicit association test. J. Pers. Soc. Psychol. 74, 1464–1480. 10.1037//0022-3514.74.6.1464 9654756

[B22] Haidt J. (2001). The emotional dog and its rational tail: a social intuitionist approach to moral judgment. Psychol. Rev. 108, 814–834. 10.1037//0033-295x.108.4.814 11699120

[B23] Haidt J. Koller S. H. Dias M. G. (1993). Affect, culture and morality, or is it wrong to eat your dog?. J. Pers. Soc. Psychol. 65, 613–628. 10.1037//0022-3514.65.4.613 8229648

[B24] Harenski C. L. Hamann S. (2006). Neural correlates of regulating negative emotions related to moral violations. Neuroimage 30, 313–324. 10.1016/j.neuroimage.2005.09.034 16249098

[B25] Hauser M. Cushman F. Young L. Jin R. K.-X. Mikhail J. (2007). A dissociation between moral judgments and justifications. Mind Lang. 22, 1–21 10.1111/j.1468-0017.2006.00297.x

[B26] Kallinen K. Salminen M. Ravaja N. Kedzior R. Sääksjärvi M. (2007). Presence and emotion in computer game players during 1st person vs. 3rd person playing view: evidence from self-report, eye-tracking and facial muscle activity data. Proc. PRESENCE 187–190.

[B27] Kaspar K. Ollermann F. Hamborg K.-C. (2011). Time-dependent changes in viewing behavior on similarly structured web pages. J. Eye Mov. Res. 4, 1–16.21603125

[B28] Kastner R. M. (2010). Moral judgments and visual attention: an eye-tracking investigation. Chrestomathy 9, 114–128.

[B29] Milgram S. (1963). Behavioral study of obedience. J. Abnorm. Psychol. 67, 371–378. 10.1037/h0040525 14049516

[B30] Navarrete C. D. McDonald M. M. Mott M. L. Asher B. (2012). Virtual morality: emotion and action in a simulated three-dimensional “trolley problem”. Emotion 12, 364–370. 10.1037/a0025561 22103331

[B31] Niedenthal P. M. Barsalou L. W. Winkielman P. Krauth-Gruber S. Ric F. (2005). Embodiment in attitudes, social perception and emotion. Pers. Soc. Psychol. Rev. 9, 184–211. 10.1207/s15327957pspr0903_1 16083360

[B32] Nosek B. A. Banaji M. (2001). The go/no-go association task. Soc. Cogn. 19, 625–664 10.1521/soco.19.6.625.20886

[B33] Olson M. A. Fazio R. H. (2009). “Implicit and explicit measures of attitudes: the perspective of the MODE model,” in Attitudes: Insights from the New Implicit Measures, eds Petty R. E. Fazio R. H. Briñol P. (New York: Taylor and Francis), 19–63.

[B34] Otero-Millan J. Troncoso X. G. Macknik S. L. Serrano-Pedraza I. Martinez-Conde S. (2008). Saccades and microsaccades during visual fixation, exploration and search: foundations for a common saccadic generator. J. Vis. 8:21. 10.1167/8.14.21 19146322

[B35] Pan X. Slater M. (2011). “Confronting a moral dilemma in virtual reality: a pilot study,” in Proceedings of the 25th BCS Conference on Human-Computer Interaction (Newcastle upon Tyne: British Computer Society), 46–51.

[B36] Partala T. Surakka V. (2003). Pupil size variation as an indication of affective processing. Int. J. Hum. Comput. Stud. 59, 185–198 10.1016/s1071-5819(03)00017-x

[B37] Patil I. Cogoni C. Zangrando N. Chittaro L. Silani G. (2014). Affective basis of judgment-behavior discrepancy in virtual experiences of moral dilemmas. Soc. Neurosci. 9, 94–107. 10.1080/17470919.2013.870091 24359489

[B38] Payne B. K. (2001). Prejudice and perception: the role of automatic and controlled processes in misperceiving a weapon. J. Pers. Soc. Psychol. 81, 181–192. 10.1037//0022-3514.81.2.181 11519925

[B39] Porter G. Starcevic V. Berle D. Fenech P. (2010). Recognizing problem video game use. Aust. N. Z. J. Psychiatry 44, 120–128. 10.3109/00048670903279812 20113300

[B40] Richeson J. A. Ambady N. (2001). Who’s in charge? Effects of situational roles on automatic gender bias. Sex Roles 44, 493–512 10.1023/A:1012242123824

[B41] Rovira A. Swapp D. Spanlang B. Slater M. (2009). The use of virtual reality in the study of people’s responses to violent incidents. Front. Behav. Neurosci. 3:59. 10.3389/neuro.08.059.2009 20076762 PMC2802544

[B42] Rudman L. A. Goodwin S. A. (2004). Gender differences in automatic in-group bias: why do women like women more than men like men? J. Pers. Soc. Psychol. 87, 494–509. 10.1037/0022-3514.87.4.494 15491274

[B43] Schwarz N. (1998). Warmer and more social: recent developments in cognitive social psychology. Annu. Rev. Sociol. 24, 239–264 10.1146/annurev.soc.24.1.239

[B44] Seidel A. Prinz J. (2013). Sound morality: irritating and icky noises amplify judgments in divergent moral domains. Cognition 127, 1–5. 10.1016/j.cognition.2012.11.004 23318349

[B45] Simpson H. M. Hale S. M. (1969). Pupillary changes during a decision-making task. Percept. Mot. Skills 29, 495–498. 10.2466/pms.1969.29.2.495 5361713

[B46] Slater M. Antley A. Davison A. Swapp D. Guger C. Barker C.. (2006). A virtual reprise of the stanley milgram obedience experiments. PLoS One 1:e39. 10.1371/journal.pone.0000039 17183667 PMC1762398

[B47] Stöber J. (1999). Die Soziale-Erwünschtheits-Skala-17 (SES-17): Entwicklung und erste Befunde zu Reliabilität und Validität. Diagnostica 45, 173–177 10.1026//0012-1924.45.4.173

[B48] Stöber J. (2001). The social desirability scale-17 (SDS-17): convergent validity, discriminant validity and relationship with age. Eur. J. Psychol. Assess. 17, 222–232 10.1027//1015-5759.17.3.222

[B49] Takushi M. (1996). “Inner mind mystique 1,” in Inner Mind Mystique [CD]. Upper Darby, PA: Relapse Records.

[B50] Thomson J. J. (1985). The trolley problem. Yale Law J. 94, 1395–1415 10.2307/796133

[B51] Valdesolo P. DeSteno D. (2006). Manipulations of emotional context shape moral judgment. Psychol. Sci. 17, 476–477. 10.1111/j.1467-9280.2006.01731.x 16771796

[B52] van Rijn H. Dalenberg J. R. Borst J. P. Sprenger S. A. (2012). Pupil dilation co-varies with memory strength of individual traces in a delayed response paired-associate task. PLoS One 7:e51134. 10.1371/journal.pone.0051134 23227244 PMC3515525

